# Changes in cellular signaling proteins in extracts from A549, H460, and U2OS cells treated with cisplatin or docetaxel

**DOI:** 10.1016/j.dib.2017.03.023

**Published:** 2017-03-18

**Authors:** Voin Petrovic, Camilla Olaisen, Animesh Sharma, Anala Nepal, Steffen Bugge, Eirik Sundby, Bård Helge Hoff, Geir Slupphaug, Marit Otterlei

**Affiliations:** a Department of Cancer Research and Molecular Medicine, Faculty of Medicine, Norwegian University of Science and Technology (NTNU), Trondheim, Norway; b The Proteomics and Metabolomics Core Facility (PROMEC) at NTNU, Faculty of Medicine, Trondheim, Norway; c Department of Chemistry, Faculty of Natural Sciences and Technology, Norwegian University of Science and Technology (NTNU), Trondheim, Norway; d Department of Chemistry and Material Technology, Faculty of Natural Sciences and Technology, Norwegian University of Science and Technology (NTNU), Trondheim, Norway

## Abstract

Cell extracts from A549, H460, and U2OS human cancer cell lines treated with cisplatin and docetaxel were analyzed by mass spectrometry (MS) proteomic analysis. The extracts were enriched for cellular signaling proteins using a mix of three different immobilized kinase inhibitors (Purvalanol B, Bisindolylmaleimide X, and (R)-3-(4-((1-Phenylethyl)amino)thieno[2,3-d]pyrimidin-6-yl)benzoic acid (SB6-060-05)) on sepharose bead columns. Raw data is deposited in the PRIDE database [1], project number PXD005286. Data presented (Table 1) shows changes relative to untreated control for each biological replicate for the three cell lines.

**Specifications Table 1**

**Table:** 

Subject area	Biochemistry, Cell Biology, Cancer Research
More specific subject area	Proteomics
Type of data	Table
How data was acquired	Mass spectrometry (MS)
Data format	Analyzed
Experimental factors	Treatment with cytostatics
Experimental features	Three human cancer cell lines
Data source location	/
Data accessibility	PRIDE database, PXD005286

**Value of the data**

1. The data is from three well studied human cancer cell lines treated with commonly used chemotherapeutics, and can therefore be used by other researchers studying these cell lines or effects of these drugs.
2. The data set describe the simultaneous pattern of change of hundreds of signaling proteins, and could be used for further modeling or network analysis.
3. Researchers studying particular signaling pathways or networks can use the data set to find information on how levels of specific proteins change upon DNA stress or between cell lines.

## 1 Data

This dataset has been produced in order to test and evaluate an updated method for targeted kinase proteomics. It contains data from MS analysis of three biological replicates of three human cancer cell lines A549, H460, and U2OS, treated with cisplatin or docetaxel, as well as their respective controls (3×3×3 samples). The raw data [1] has been analyzed and expressed as the percent of control. The data has also been analyzed using the Wilcoxon signed rank test [2] to search for significant changes across all three replicates of a particular protein. The detailed findings are published elsewhere [3].

## 2 Experimental design, materials and methods

### 2.1 Cell lines

H460 (NCI-460; large cell lung cancer) was grown in RPMI-1640 medium (Sigma-Aldrich). The cell lines A549 (ATCC CCL-185; non-small cell lung cancer) and U2OS (ATCC HTB-96; osteosarcoma) were grown in DMEM (Sigma-Aldrich). Both media were supplemented with fetal bovine serum (10%), amphotericin B (2.5 µg/mL), l-glutamine (2 mM, all from Sigma-Aldrich) and penicillin (100 units/mL)-streptomycin (0.1 mg/mL) (Gibco, NY, USA). Cells were cultivated in a humidified atmosphere (95% air, 5% CO_2_, 37 °C).

### 2.2 Cell treatment and lysis

Cells were treated with docetaxel (5 ng/mL) or cisplatin (5 µM for A549 and U2OS, 0.5 µM for H460, close to IC_50_ at day 4) for 24 h and harvested by scraping in ice-cold PBS. The cells were washed once, pelleted, snap-frozen in liquid nitrogen and stored at −80 °C until use. For the lysis, the samples were thawed and mixed with 3×packed cell volume of lysis buffer (50 mM HEPES-NaOH pH 7.5, 0.5% Triton X-100, 150 mM NaCl, 1 mM EDTA, 1 mM EGTA, 10 mM NaF, 2.5 mM Na_3_VO_4_ (sodium orthovanadate), and 1X Halt^™^ protease and phosphatase inhibitor cocktail (ThermoFisher Scientific, USA)). Samples were sonicated for 2 min on ice prior to centrifugation (15 min, 14,000 rpm, 4 °C) and the supernatant collected for further analysis.

### 2.3 Enrichment of signaling proteins and mass spectrometer (MS) analysis

A modified multiplexed inhibitor bead (MIB) assay was used to enrich the cell lysates for kinases and other signaling proteins [3]. After that the samples were analyzed using an EASY-nLC 1000 UPLC system (Thermo Scientific, USA) interfaced with a Q Exactive mass spectrometer (Thermo Scientific, USA) via a nanospray ESI ion source (Thermo Scientific, USA). 10 μL of the peptide solution was injected onto an Acclaim PepMap100 C-18 trap column (75 μm i.d., 2 cm, C18, 3 μm, 100 Å, Thermo Scientific, USA) and an acclaim PepMap100 C-18 analytical column (75 μm i.d., 50 cm, 2 μm, 100 Å, Thermo Scientific, USA) using a 120 min multi-step gradient (5 min 2%–6% B, 26 min 6%–12% B, 49 min 12%–20% B, 15 min 20%–28% B, 7 min 28%–40% B, 3 min 40%–100% B, 15 min at 100% B; where B is 0.1% formic acid in acetonitrile and A is 0.1% formic acid in water) at 250 nL/min. Peptides were analyzed in positive ion mode under data dependent acquisition (DDA) using the following parameters: Electrospray voltage 1.9 kV, HCD fragmentation with normalized collision energy 30, automatic gain control target value of 3×10^6^ for Orbitrap MS and 1×10^5^ for MS/MS scans. Each MS scan (400–1600 m/z) was acquired at a resolution of 70,000 FWHM, followed by 10 MS/MS scans with isolation window 4.0 m/z, triggered for intensities above 1.7×10^4^, at a maximum ion injection time of 100 ms for MS and 60 ms for MS/MS scans. A dynamic exclusion of 40 s was used as well as charge exclusion for unassigned, 1, and greater than 4. Thermo Sieve ™ was used to align MSMS spectra. Preview 2.3.5 (Protein Metrics Inc.) was used to determine optimal search criteria [4]. These were plugged in Max Quant v 1.5.30 [5] mapping the spectra over Human canonical proteome with isoforms (Uniprot March 2016) [6]. The following search parameters were used: enzyme specified as trypsin with maximum two missed cleavages allowed; deamidation of asparagine/glutamine, oxidation of methionine, N-terminal acetylation, and dimethylation of lysine/arginine as variable modifications. Precursor mass tolerance was set to 20 ppm with fragment mass tolerance of 0.02 Da. False discovery rate was set to 0.01 (high confidence) for peptide as well as protein group identification. Label free quantification (LFQ) algorithm [7] was used to estimate the protein amounts in the sample using match between the runs with alignment time of 20 min and match time window of 1 min. This option was disabled for comparing three technical replicates. These LFQ values were log transformed with base 2 and the transformed control values were subtracted. The resulting values reflecting the change relative to control for each condition were subjected to two sided non-parametric Wilcoxon Sign Rank Test [2] as implemented in MATLAB R2015a (MathWorks Inc.) in order to check the consistency in directionality of the change, namely a negative sign reflecting decreased and positive sign reflecting increased expression of respective protein group. The choice of this non-parametric test avoids the assumption of certain type of null distribution as in Student׳s *t*-test by working over the Rank of the observation instead of observation value itself. Furthermore, it also makes it robust to outliers and extreme variations noticed in observed values.

## References

[1] Vizcaíno J.A., Csordas A., del-Toro N., Dianes J.A., Griss J., Lavidas I., Mayer G., Perez-Riverol Y., Reisinger F., Ternent T., Xu Q.W., Wang R., Hermjakob H. (2016). update of the PRIDE database and related tools. Nucleic Acids Res..

[2] Gibbons J.D., Chakraborti S. (2010). Nonparametric Statistical Inference.

[3] V. Petrovic, C. Olaisen, A. Sharma, A. Nepal, S. Bugge, E. Sundby, B.H. Hoff, G. Slupphaug, M. Otterlei, On-column trypsinization allows for re-use of matrix in modified Multiplexed Inhibitor Beads assay, Anal. Biochem., 523, 2017, 10–16. 10.1016/j.ab.2017.01.027. 10.1016/j.ab.2017.01.027 28167071

[4] Kil Y.J., Becker C., Sandoval W., Goldberg D., Bern M. (2011). Preview: a program for surveying shotgun proteomics tandem mass-spectrometry data. Anal. Chem..

[5] Cox J., Mann M. (2008). MaxQuant enables high peptide identification rates, individualized p.p.b.-range mass accuracies and proteome-wide protein quantification. Nat. Biotechnol..

[6] Boutet E., Lieberherr D., Tognolli M., Schneider M., Bansal P., Bridge A.J., Poux S., Bougueleret L., Xenarios I., Edwards D. (2016). UniProtKB/Swiss-Prot, the manually annotated section of the uniprot knowledgebase: how to use the entry view. Plant Bioinformatics: Methods and Protocols.

[7] Cox J., Hein M.Y., Luber C.A., Paron I., Nagaraj N., Mann M. (2014). Accurate proteome-wide label-free quantification by delayed normalization and maximal peptide ratio extraction, termed MaxLFQ. Mol. Cell. Proteom..

