# Supplementary material for: Changes in cellular signaling proteins in extracts from A549, H460, and U2OS cells treated with cisplatin or docetaxel
Source: Data Brief. 2017 Mar 18;12:18–21. doi: 10.1016/j.dib.2017.03.023 (PMC5362150; doi:10.1016/j.dib.2017.03.023)
Supplement: Supplementary file 2 — Supplementary material [file mmc1.docx]

The other authors declare no conflicts of interest.

“Conflict of Interest is mandatory for revision, so while submitting please submit the file by selectin the description from the drop down. Please click here to download the Conflict of Interest form.”

We could not find any conflict of interest form to download, the link on your webside did not work.
